# COVID-19 and the UK Biobank—Opportunities and Challenges for Research and Collaboration With Other Large Population Studies

**DOI:** 10.3389/fcvm.2020.00156

**Published:** 2020-08-27

**Authors:** Mohammed Y. Khanji, Nay Aung, C. Anwar A. Chahal, Steffen E. Petersen

**Affiliations:** ^1^Barts Heart Centre, Barts Health NHS Trust, London, United Kingdom; ^2^NIHR Barts Biomedical Research Centre, William Harvey Research Institute, Queen Mary University of London, London, United Kingdom; ^3^Department of Cardiology, University of Pennsylvania, Philadelphia, PA, United States; ^4^Department of Cardiovascular Diseases, Mayo Clinic, Rochester, NY, United States

**Keywords:** COVID-19, UK Biobank, population studies, precision medicine, epidemiology

## Abstract

Large population studies such as the UK Biobank provide great opportunities for understanding the pathophysiology, health impact and prognostic factors associated with COVID-19, a condition that has had significant impact on almost everyone around the world. We highlight the vast opportunities, challenges and limitations for research and collaboration from the UK Biobank and other large population studies in helping us better understand and manage both current and potential future pandemics.

## Background

The Coronavirus disease 2019 (COVID-19) pandemic has had a profound impact on health and the way people live globally. Our knowledge of the disease is increasing at a fast pace and thus far has largely been from observational studies and registries (1, 2), with an increasing number of clinical trials underway assessing treatment options, vaccination and other preventative strategies to limit the morbidity and mortality associated with it (www.covid-trials.org).

There have been reports that the disease has worse outcomes in those who are older, have cardiovascular disease, and may potentially be linked to certain medications, as well as socially disparate groups. The studies to date, whilst essential given the extraordinary circumstances, are prone to potential limitations inherent in clinical observational studies that generally lack systematic assessment and initially included mostly those who had been moderate or severely affected by COVID-19 and thus required hospitalization (3). The main presentations have been with cough and fever and confirmed cases were initially based on positive nasal and throat swabs for SARS-CoV-2 leading to respiratory failure. Oxygen support, non-invasive or invasive ventilation have been the main stay of treatment to date with reports of propensity to thromboembolic complications and potential cardiac manifestations (4).

## Large Population Studies

Large longitudinal population studies provide a powerful way of tracking the health of a large group of the population over time (5). The impact of factors such as environmental, genetic and lifestyle choices on health and outcomes can be assessed to enable researchers to better understand the drivers for health and potential differences between groups of people. With the ultimate aim of improving health through public health policies and their delivery. A number of large population studies are under way around the world including the UK Biobank study, the China Kadoorie Biobank, USA Million Veteran Program, and the Prospective study of 500,000 adults in Chennai, India (6–9). Each study will have variations in the number of people enrolled, although these specific ones aim to involve between 500,000 or more adults. Each study varies in the populations enrolled (including age and ethnicity) and extent of factor measurement (imaging and genetic testing, for example). For the purpose of this manuscript we will discuss the UK Biobank study and other population studies to assess the opportunities and challenges in relation to the recent COVID-19 pandemic.

### UK Biobank Cohort Study

The UK Biobank is a prospective cohort study with deep phenotype and genotype data collected for over 500,000 individuals aged between 40 and 69-years-old at recruitment between 2006 and 2010, from across England, Scotland and Wales (6). The rich dataset contains biological measurements, lifestyle questionnaires and health-related information, blood and urine biomarkers for all participants. Genome-wide genotype data collected on all participants are providing opportunities for genetic association discoveries and genetic basis of complex traits that could guide future therapeutic targets (10).

Additional information in a large subset are available or in the process of being collected, such as deep imaging (MRI of the heart, brain and abdomen, carotid ultrasound scanning and bone densitometry) in 100,000 with a target completion in 2023 (11, 12). Almost half of these participants have already been scanned. There is also funding confirmed to allow follow-up scanning in about 10,000 of these volunteers.

The number of UK Biobank participants scanned pre-COVID was under just below 50,000. The imaging centers stopped scanning participants on the 13th March due to COVID-19 and will resume scanning when deemed safe. Although only a 1/5 of the UK Biobank are planned to have imaging, it still provides detailed imaging information on 100,000 individuals which is substantial and unprecedented for any national biobank. Another advantage is the on-going rescanning effort which will enable the assessment of pre- and post-COVID changes.

Follow-up health information is provided by robust linkage to primary care electronic health records, death and cancer registries and hospital admission records. With increasing outcome information generated over time the epidemiological opportunities of the UK Biobank study will be vast.

The open source nature of the UK Biobank study is novel and therefore allow any researcher to benefit from the size and scope of the study through an application process. This is particularly commendable given longitudinal studies are notoriously expensive and logistically challenging to execute.

### UK Biobanks and COVID-19

**Figure 1:**
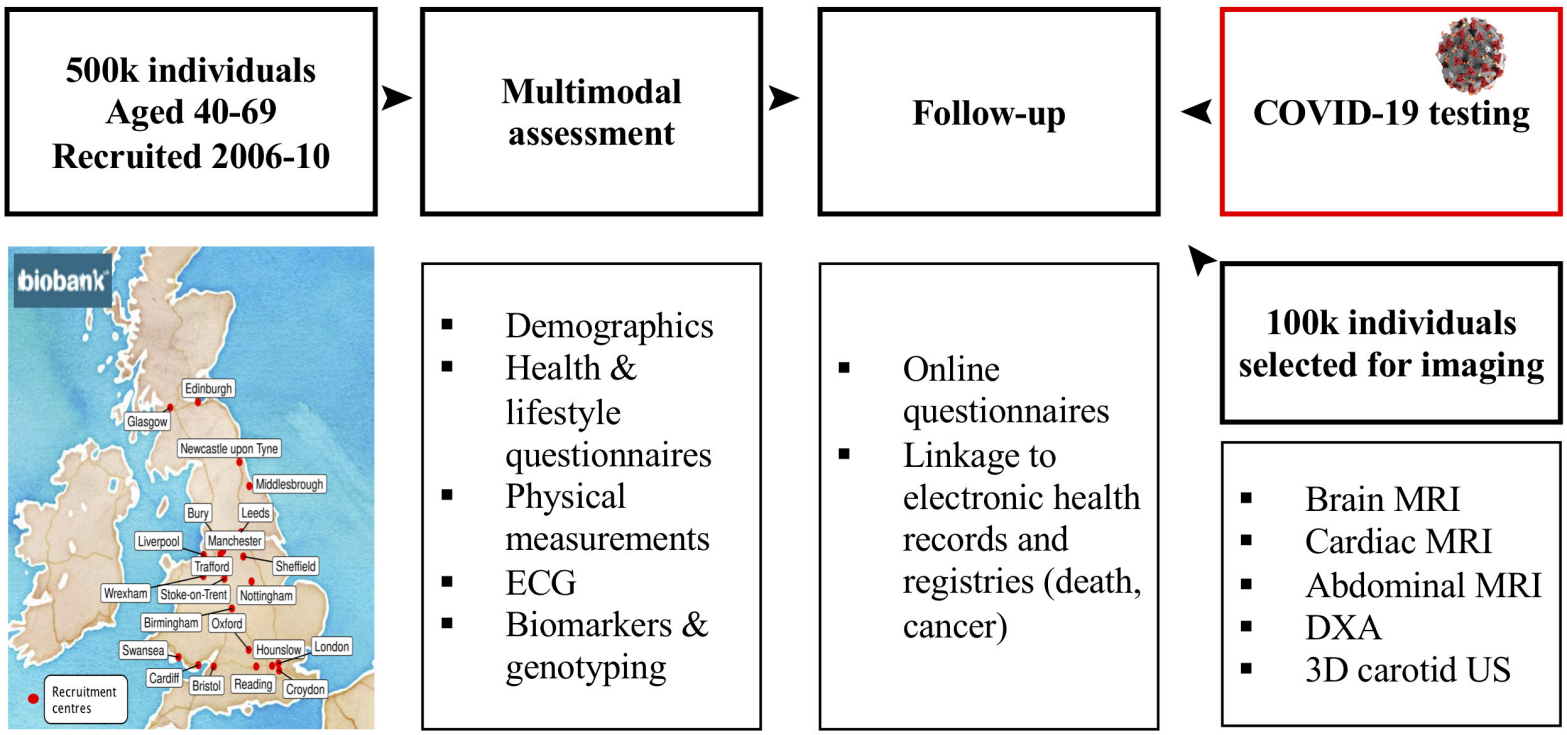
Overview of the UK Biobank Study—A unique multimodal population study of 500,000 with recent addition of COVID-19 related data.

With the COVID-19 pandemic affecting so many people, the UK Biobank study provides great opportunity for epidemiological analysis and allow us to explore characteristics that are associated with poorer outcomes in COVID-19 patients along with those that may be protective. The association of lifestyle, comorbidities, medication and phenotypic information with outcomes will become an invaluable source as more data becomes available on those that are tested for presence of COVID-19, especially as the UK government plans to ramp up targets for testing in the general population and not just those admitted to hospital or health care workers (Figure 1).

Results of COVID-19 tests for UK Biobank participants are provided by Public Health England for participants residing in England. These are being updated on a weekly basis and include both positive and negative test results. On a monthly basis, information directly linked to primary care data, hospital inpatient data, and death data will be made available along with critical care data for those individuals that have been confirmed as having COVID-19. Table 1 provides examples of large population studies and which studies are actively collecting COVID-19 related information. Even at the time of revising the manuscript for the journal it was clear that a large number of the Biobanks were taking active steps in increasing the COVID-19 related data to help us better understand the disease.

**Table 1:** Overview of Large Biobanks around the world and where COVID-19 related data is already becoming available.

**Country**	**Year enrolled**	**Number enrolled**	**Additional ongoing** **Enrolment**	**Variables collected**							**SARS-CoV-2 information being made available**	**Funding**	**Additional information**
				**Biological measures**	**Surveys**	**Blood**	**Urine**	**Stool**	**Scalp hair**	**Genetics**			
**UK Biobank**	2007–2010	500k	Subset of 100k with imaging data (2014–2023) Subset 50k exercise stress test with ECG Subset 100k with Activity monitor (2013–2015)	Anthropometrics Blood pressure Lung vital capacity Bone density Intra ocular pressure	Self-completed lifestyle and general health	+	+	–	–	500k with microarray 50k WES (planned 130k 2020) WGS planned	Yes COVID-19 test results (hospitalized and public screening) with linking to electronic health records. Tested positive −1,150, tested negative 6,118 (June 2020) Serial antibody testing in 20k participants and invitation to their children and grandchildren underway	UK DoH MRC Wellcome Trust £62M to date	Phased releases for imaging, WES, WGS data. Primary care data, Hospital-linked admissions Cancer registry data linked Death status linked
**China Kadoorie Biobank**	2004–2008	510k 30–79 y/o	Subgroup 25k tested every few years	Baseline clinical variables	Medical and lifestyle	+	–	–	–	~100k with candidate array (384 SNPs) Up to ~100k with GWAS array (700k SNPs)	No	Chinese government	Joint venture between University of oxford and Chinese Academy of Medical Sciences 8 years follow-up data available
**China Taizhou** **Biobank**	2004	100k	Planned	Anthropometrics Tissue Disease-oriented	Interviewer-conducted surveys	+	–	–	–	Unknown	No	Chinese government	Fudan University Institute of Health Sciences Includes CSF, frozen tissue, FFPE
**National Cancer Tissue Biobank** Chennai, India^*^	500,000			Cancer biobank							–		Public-private partnership
**USA** **Million Veteran Program**	2011 825k	Target 1M	–	EHR	Self-completed Lifestyle Health	+	–	–	–	GWAS array WES WGS	Yes Questionnaire on participants physical and mental health and experiences underway (June 2020) Summary data on COVID-19 deaths, active and convalescent cases	DoH	United States Department of Veterans Affairs
**USA All of Us**	2015	~350k to date	Goal to target 1M by 2022	Anthropometrics EHR	Self-completed questionnaires	+	+	–	–		Yes Antibody testing planned in 10,000 COVID-19 Questionnaire on participants physical and mental health and experiences underway Collection of relevant electronic health record data from >200k participants	NIH Google Verily life Sciences	Was built on the NHGRI “The American project,” launched as “Precision Medicine Initiative” 2016 and renamed to “All of Us” in 2016
**Lifelines Cohort Study** **Netherlands**	2006–2013	167k 25–50 y/o and 3 generations invited (includes offspring, partners and parents	Add on studies reviewed on request e.g., Omics profiling agreed in subset of 10k	Anthropometrics Blood pressure ECG Lung vital capacity Cognitive	Lifestyle Health Personality Work Living environment	+	+	+	+	GWAS array completed Planned microbiome	Yes COVID-19 Questionnaires completed by > 70,000 participants on physical and mental health and experiences (June 2020)		30-year longitudinal study
**deCODE** **Iceland**	1996-present	230k to date	Planned enrolment entire Icelandic population of 364k	Medical records Genealogical records	Unknown	+	–	–	–	GWAS array (337k) WES WGS (15k)	Yes, 9,199 individuals invited (symptomatic and their contacts), 1,221 (13.3%) positive for SARS-CoV-2 10,797 population volunteers (0.8% positive) + Randomly selected screening of 2,283 (0.6% positive) Virus sequenced from 643 individuals (April 2020)	Private initiative Amgen	
**Finland Fingen**	2018–2024	Planned 500k	No	EHR		+	–	–	—	GWAS arrary (500k)	Yes 264 cases tested positive for COVID-19 (June 11 release) 203,376 populations controls	Finnish Universities and Private partners	Private includes pharmaceutical companies 230k samples collected to date

DoH, Department of Health; ECG, electrocardiograph, FFPE, formalin-fixed, paraffin-embedded; GWAS, genome wide association studies; k, Thousand; NHGRI, National Human Genome Research Institute; SNP, single nucleotide polymorphisms; WES, whole exome sequencing; WGS, whole genome sequencing; y/o, Years-old;

* *limited information available*.

The UK Biobank for example has now also initiated a coronavirus antibody study where they will invite a representative sample of 20,000 of the total 500,000 participants who express an interest in participation. They will be asked to self-collect 0.5 mls of blood from finger prick for antibody testing. This will be repeated monthly for at least 6-months. Children and grandchildren of the participants, who are over the age of 18 years will also invited to provide blood samples for both antibody testing and genetic testing to additionally assess for genetic susceptibility in young adults.

## Opportunities

The growing COVID-19 related information for a cohort with a rich phenotype and genotype assessment along with regular outcome measure updates will allow researchers to define the relevance of wide-ranging genetic and non-genetic factors to severity and outcomes based on age, lifestyle, co-morbidities, prescribed medications, environmental, and regional factors. The outcome data now and in the future will provide a comprehensive analysis of the mortality rates and associated morbidity in the UK cohort. Particularly where the data are able to help identify risk factors that predispose to poorer outcomes and those that could be protective thus guiding lifestyle and prevention recommendations. This creates a colossal opportunity for detailed analysis of the cohort and the impact of the disease on longer term health and well-being of survivors that will guide future research and public health policies.

In those who have already undergone deep imaging phenotyping, follow-up scanning will provide novel insights in understanding the downstream, long-term effects of COVID-19 exposure on biological systems. Analysis of the subset of participants undergoing follow-up imaging could also provide better understanding of pathophysiology using the pre- and post-COVID-19 imaging data.

The UK Biobank is already one of the largest contributors to an international consortium to investigate the genetic determinants of vulnerability to COVID-19, disease severity and outcomes (https://www.covid19hg.org). The second-round meta-analyses of the genome-wide association studies of COVID-19 status had been released. This initiative may not only enrich our knowledge of COVID-19 biology but provide the genetic evidence for drug targets and assist in the development of genetically informed risk assessment of COVID-19 susceptibility. The genetic data also allow the conduct of Mendelian randomization studies which permit evaluation of causality in observational settings (13).

## Challenges and Limitations and Future Perspectives

There is already a large interest from researchers globally in the UK Biobank study which will lead to healthy competition for research and publication. As large groups of researchers may be working in silos on similar projects there may large efforts with those being quickest getting publications. Due to the need for timely submissions for publication there is a potential risk for less rigor or quality control checks during data cleaning and analysis (14).

The UK Biobank enrolled middle and older aged adults only and Caucasians making up the vast majority of participants, with limited number of other ethnicities (15). No participants were under the age of 40 at enrolment 16 years ago. Thus, only those who are about 56 years and older at the time of the COVID-19 pandemic are included. The recently proposed inclusion of children and grandchildren of participants for antibody testing will partly reduce this limitation. There is also evidence of healthy-volunteer bias in the UK Biobank cohort. Therefore, although the UK Biobank data are valid for the investigation of biological associations given its large sample size and the heterogeneity of measurements, it cannot be used to ascertain true disease prevalence in the population (16).

Impact of delayed uptake of population screening through swabbing in the UK in those with milder disease along with lack of systematic symptom data may limit the research potentials. There is also a chance that key findings may only be generated once we have passed the worst period of the pandemic.

Data sharing that allow combination of large cohorts from around the world including the UK Biobank study and other larger population initiatives will increase the richness of the data and allow better assessment of geographical variations, ethnic differences and similarities to better guide public health policies and ways of managing future pandemics.

## Conclusions

COVID-19 has had a global impact and will change our health care approaches in the future. The UK Biobank population study can offer great opportunities given the detailed systematic nature of the assessments along with the growing linkage to the current COVID-19 testing and outcome data. The true potentials of the UK Biobank and other large population-based research studies will become evident as the data accumulate over time and may be enhanced further by linking large population-based studies which can allow limitations such as ethnic and geographical differences and guide optimisation of public health policies.

## Data Availability Statement

Publicly available data from the UK Biobank study was analyzed in this study. The datasets are available to researchers through an open application via https://www.ukbiobank.ac.uk/register-apply/.

## Author Contributions

All authors have made substantial contributions to conception and design, involved in drafting the manuscript or revising it critically for important intellectual content, and given final approval of the version to be published. All authors had full access to all the data in the study and take responsibility for the integrity of the data and the accuracy of the data analysis.

## Conflict of Interest

SP provides consultancy and has stock options for Circle Cardiovascular Imaging, Inc., Calgary, Alberta, Canada. The remaining authors declare that the research was conducted in the absence of any commercial or financial relationships that could be construed as a potential conflict of interest.
